# Targeted incorporation of collagen IV to the basement membrane: A step forward for developing extracellular protein therapies

**DOI:** 10.1016/j.jbc.2025.110384

**Published:** 2025-06-14

**Authors:** Elena N. Pokidysheva, Sara F. Tufa, Douglas R. Keene, Billy G. Hudson, Sergei P. Boudko

**Affiliations:** 1 Division of Nephrology and Hypertension, Department of Medicine, Vanderbilt University Medical Center, Nashville, Tennessee, USA; 2 Center for Matrix Biology, Vanderbilt University Medical Center, Nashville, Tennessee, USA; 3 Aspirnaut Program, Vanderbilt University Medical Center, Nashville, Tennessee, USA; 4 Micro-Imaging Center, Shriners Children's, Portland, Oregon, USA; 5 Department of Biochemistry, Vanderbilt University, Nashville, Tennessee, USA; 6 Department of Pathology, Microbiology and Immunology, Vanderbilt University Medical Center, Nashville, Tennessee, USA; 7 Vanderbilt-Ingram Cancer Center, Vanderbilt University, Nashville, Tennessee, USA; 8 Vanderbilt Institute of Chemical Biology, Vanderbilt University, Nashville, Tennessee, USA; 9 Department of Biological Sciences, Vanderbilt University, Nashville, Tennessee, USA; 10 Department of Cell and Developmental Biology, Vanderbilt University, Nashville, Tennessee, USA

**Keywords:** basement membrane, collagen IV, NC1 domain, Alport syndrome, Gould syndrome, molecular orthotics

## Abstract

The collagen IV scaffold serves as a fundamental structural unit of the basement membrane (BM). Understanding its structure, assembly, and function is essential for tissue engineering, the design of organoid models, and developing therapies for diseases such as Alport syndrome, Gould syndrome, psoriasis, eye abnormalities, hearing loss, and others, where collagen IV is required for structural integrity and functionality of the BM. The collagen IV molecule is a 400 nm long heterotrimer, comprising non-collagenous 1 (NC1), collagenous, and 7S domains. The assembly of the collagen IV scaffold involves oligomerization of the C-terminal NC1 and the N-terminal 7S domains, along with lateral associations within the collagenous domain. However, the detailed architecture and assembly mechanisms of the collagen IV scaffold remain unclear. Here, we investigated the potency and mechanism of recombinant single-chain NC1 trimer incorporation into the collagen IV scaffold. We discovered that the NC1 trimer influences the overall assembly of the basement membrane by affecting the quality of the developing collagen IV scaffold in a dose-dependent manner, without impacting already established scaffolds. This interference occurs through the hexamerization of supplemented NC1 trimers with endogenous NC1 domains, as the NC1 trimer becomes sulfilimine crosslinked with the existing chains. Overall, the single-chain NC1 trimer of collagen IV is crucial for developing novel extracellular therapies in two main ways: (1) facilitating the delivery and incorporation of functional replacements like collagen IV fragments and (2) inhibiting the formation of new basement membranes in conditions such as tumor growth and detrimental vascularization.

Collagen IV has been a subject of research for over half a century. Still, only recently have we appreciated its role in evolution, development, and function in animals as multicellular organisms (1, 2). Besides being a structural building block for all basement membranes that organize tissues and organs, it serves as a scaffold for binding multiple molecules to signal and guide cell behavior during organism development and lifelong function (1, 3, 4, 5, 6, 7, 8, 9). Collagen IV-related basement membrane (BM) pathologies include Alport syndrome, Gould syndrome, HANAC, Goodpasture disease, thin basement membrane disease, psoriasis, eye abnormalities, hearing loss, *and so on* (10, 11, 12, 13, 14, 15, 16, 17, 18, 19, 20, 21, 22). It is imperative to understand the structural organization and assembly mechanisms of collagen IV scaffolds to rationally develop therapies for basement membrane quality, composition, and growth regulation.

In mammals, there are six alpha chains of collagen IV (α1–α6) that form heterotrimeric protomers with a selective stoichiometry of α1α2α1, α3α4α5, and α5α6α5 (23). Our knowledge about collagen IV scaffold assembly is based on studies of the predominant and primordial α1α2α1 composition (2). Once secreted, collagen IV α1α2α1 protomers are exposed to high chloride concentration (24) which triggers the complexation of two trimers through the carboxy-terminal non-collagenous (NC1) domains (25, 26). Maintaining the chloride pressure outside the cell is imperative to ensure the stability and conformational integrity of the NC1 hexamer (27, 28). The 7S domain, located on the opposite end of the protomer, oligomerize into a tetramer of trimers (29, 30, 31). These two interactions along with lateral aggregation of protomers are considered key for the scaffold assembly, integrity, and stability (30, 32).

The NC1 domain is a primordial unit of collagen IV that harbors coding features for chain selection and trimer assembly inside the cell and hexamer assembly outside the cell (4, 33, 34, 35, 36, 37). We recently discovered that the NC1 trimers are targeted to distant tissue locations in *Drosophila* (38). Although NC1 is only one-seventh of the whole collagen IV molecule, it bears sufficient information for homing it to the BM. It provides an initial clue about using NC1-containing fragments of collagen IV for developing protein replacement therapy in humans (39). Here we tested a newly invented tool, a single-chain NC1 trimer (a single polypeptide chain that comprises the sequences of the NC1 domains of the α1, α2, and α1 chains of collagen IV) (26), for its capacity to interact with the mammalian basement membrane as a potential therapeutic agent or carrier. Indeed, it gets incorporated into the basement membrane *via* oligomerization with the endogenous NC1 domains of full-length collagen IV protomers. Moreover, the NC1 trimer interferes with the assembly process in a dose-dependent manner and, at excessive concentrations, inhibits collagen IV scaffold assembly and disrupts the overall basement membrane integrity. This proof-of-concept study positions the recombinant NC1 trimer of collagen IV as a potential therapeutic intervention for different pathologic conditions ranging from collagen IV deficiency to excessive neoformation of the basement membrane.

This article is part of a series of publications on the biology of collagen IV in the *Journal of Biological Chemistry*. In previous articles, we reported a novel method for the recombinant production of functional collagenous fragments, allowing for controlled chain composition and register (40). We explored the essential role of chloride pressure in the assembly and stability of the collagen IV scaffold (24) and uncovered the mechanism behind collagen IV assembly in *Drosophila* (38). Our findings revealed that the NC1 trimer contains sufficient information for the distant delivery and incorporation into a developing basement membrane in *Drosophila* (38). Additionally, we elucidated the origin of a kidney-specific isoform of collagen IV, which enables compaction of the kidney filter (36). We analyzed the evolution of collagen IV genes and discovered that the presence of multiple cysteine residues in the collagenous domain of the kidney-specific isoform confers additional mechanical strength to GBM to withstand high hydrostatic pressure (2). The current study demonstrates that the NC1 domain can effectively integrate into the developing endogenous collagen IV scaffold in mammalian cell cultures, providing insights for developing protein replacement therapies for Alport and Gould syndromes.

## Results

### Single-chain NC1 trimer oligomerizes with native collagen IV protomers in solution

Our recently invented single-chain NC1 trimer (a single polypeptide chain that comprises the sequences of the NC1 domains of the α1, α2, and α1 chains of human collagen IV) demonstrated natural activity toward the formation of the NC1 hexamer and was also able to co-assemble into a heterotypic hexamer with tissue-derived NC1 monomers (26) thus demonstrating a potential to co-assemble with native protomers of collagen IV.

**Figure 1:**
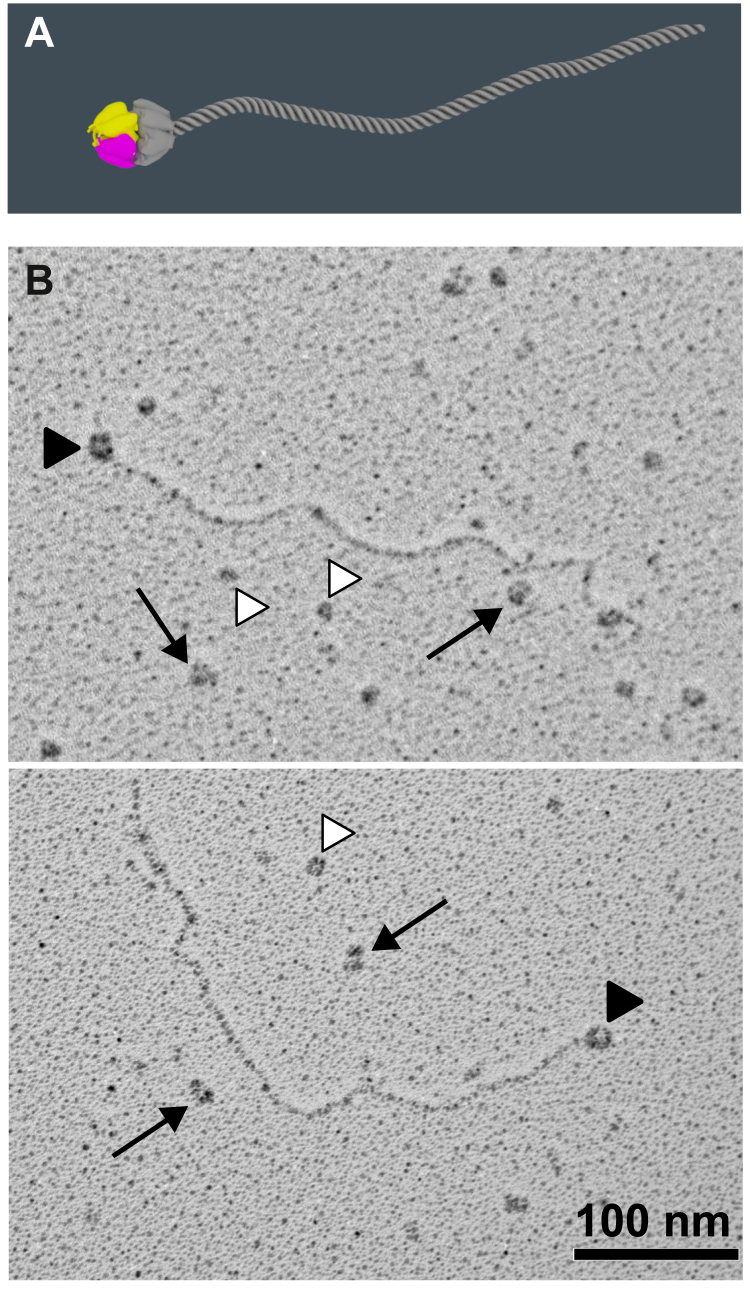
**The NC1 trimer associates with the endogenous full-length collagen IV protomer.***A*, schematic drawing of the recombinant NC1 trimer (colored) assembled with the native full-length collagen IV protomer (*gray*). *B*, chloride-free PFHR-9 conditioned media was pulled down using anti-FLAG resin and analyzed by rotary shadowing EM. The NC1 trimer pulls full-length collagen IV protomers. *Black arrows* point to self-assembled recombinant NC1 trimers into hexamers. *Black arrowheads* point to NC1 hexamers assembled from recombinant and protomer trimers. *White arrowheads* point to recombinant NC1 trimers.

Here we demonstrate that the NC1 trimer can also oligomerize with the full-length type IV collagen protomers in solution. For this experiment, the recombinant NC1 trimers were incubated with conditioned media of PFHR-9 cells containing secreted collagen IV protomers, and then the NC1 trimer was pulled down using an anti-FLAG resin. The pulled fraction was then analyzed by rotary shadowing EM (Fig. 1). Full-length collagen IV protomers were observed. Therefore, the NC1 trimer is competent to oligomerize with the full-length collagen IV protomers *via* NC1 hexamerization.

### Setup to study the effect of the exogenous NC1 domains on BM and collagen IV assembly

**Figure 2:**
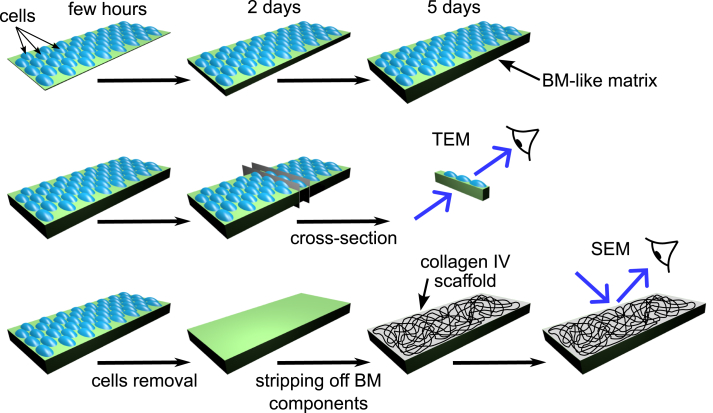
**Experimental setup to study basement membrane in PFHR-9 cell culture.** PFHR-9 cells were grown to confluency in the *high* glucose DMEM medium and then medium was switched to the *low* glucose DMEM containing 50 μg/ml of ascorbic acid. The cross-sections were analyzed by transmission electron microscopy (TEM). n another set of experiments cells were removed with lysis buffer and deposited matrix was washed with EDTA-containing buffer to remove most BM component but not collagen IV. The resulting surface of stripped collagen IV scaffold was imaged using scanning electron microscopy (SEM).

The PFHR-9 mouse endodermal cell line has been an experimental model for studying BM and collagen IV biology for over 40 years (25, 41, 42, 43, 44, 45, 46, 47). We used PFHR-9 cell culture to assess the quality of the deposited BM and collagen IV scaffold using transmission and scanning electron microscopy (EM) (Fig. 2).

**Figure 3:**
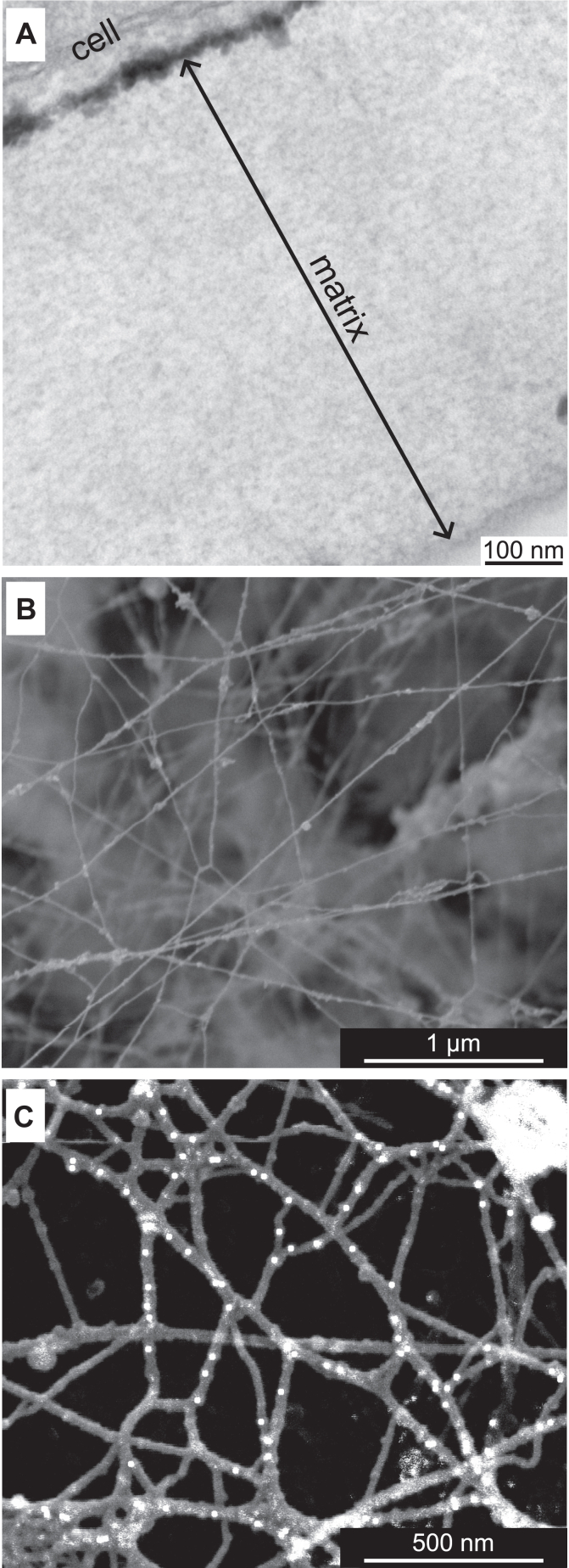
**Collagen IV scaffold deposited by PFHR-9 cell culture.***A*, transmission EM analysis of the PFHR-9 model basement membrane. *B*, scanning EM of the collagen IV scaffold after removal of cells and other components than collagen IV BM. *C*, immuno-gold labeling of type IV collagen NC1 domain analyzed by scanning EM.

While transmission EM was used for imaging overall BM (Fig. 3*A*), scanning EM methodology was developed for imaging collagen IV scaffold (Fig. 3*B*).

In brief, we lysed the cells with a mild detergent, and most of the BM proteins, but collagen IV, were dissolved and washed away by treating the deposited matrix with a high concentration of EDTA. The exposed collagen IV scaffold can be visualized by scanning EM either directly (Fig. 3*B*) or after immuno-labeling with an antibody (Fig. 3*C*). The architecture of the collagen IV scaffold aligns with previous studies of tissue BMs (48, 49, 50).

We estimated the number of collagen IV protomers within an average strand of the network using two different approaches. First, we calculated the average density of immuno-gold labels per unit length of the filament. By dividing the total number of gold particles observed in the image by the total length of the filaments, we found an average of 5.3 gold particles per 400 nm length of the individual protomer. Therefore, each average filament in the network consists of approximately five to six protomers. In the second approach, we measured the nearest neighbor distances between the immuno-gold particles. This method was applied to images at two different magnifications, and both yielded similar results. The average distance between the nearest immuno-gold particles was 59.5 nm. This value is lower than the 100 nm reported for the *in vitro* self-assembly studies (32, 51). One possible explanation for this discrepancy is an intertwining of the collagenous domain around one or more protomers *in vivo,* which can also be facilitated by other matrix proteins. Based on our findings, we conclude that 5 to 7 protomers associate laterally to form strands or filaments that make up the collagen IV network. This conclusion is consistent with previous seminal studies of basement membrane structure, which have demonstrated the lateral association of collagen IV protomers into strands (52, 53).

### The NC1 trimer, but not the hexamer, disrupts the assembly of the model basement membrane

**Figure 4:**
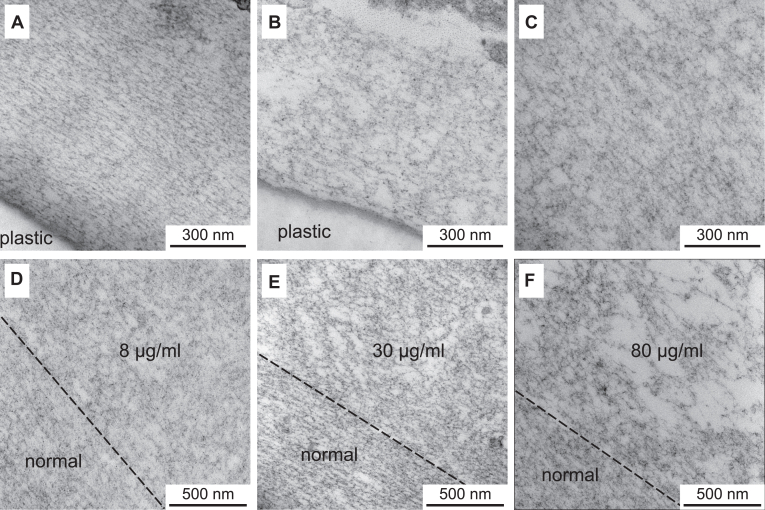
**The NC1 trimer disrupts the organization of the model basement membrane.***A*, cross-section of the model basement membrane grown under normal conditions. *B*, matrix of the PFHR-9 cells stably transfected with a GFP-containing version of the single-chain NC1 trimer showing significant disruption of the matrix. *C*, matrix deposited by regular PFHR-9 cells grown in the presence of 80 μg/ml of the NC1 trimer. *D*–*F*, concentration-dependent effect of the presence of the NC1 trimer in the medium on disruption of the basement membrane deposited by the PFHR-9 cells. Cells were initially grown under normal conditions for 3 days, and then the NC1 trimer was added to the medium for three more days at various concentrations. The degree of disruption correlates with increasing concentration of the added protein.

We generated a stable line of PFHR-9 expressing a GFP-containing version of the single-chain NC1 trimer. We found that deposited BM is significantly distorted when compared to a regular PFHR-9 cell line (Fig. 4, *A* and *B*). Next, we have examined whether supplementation of the recombinant NC1 trimer to the growth media of regular PFHR-9 cells would have a similar effect on the overall structure and integrity of the model basement membrane. Figure 4*C* shows the transmission EM image of the matrix cross-section after growing the PFHR-9 culture in the presence of 80 μg/ml of the NC1 trimer in the medium. The matrix is disorganized in a similar way. There are holes and randomly oriented strands in the affected matrix as opposed to the regular and oriented structure of the control. No difference is observed when the NC1 trimer is added to the medium or co-expressed with the endogenous collagen IV. It can therefore be concluded that crucial steps of the collagen IV scaffold assembly happen outside the cell and extracellular supplementation of the NC1 domain has sufficient effect.

To test the effect of concentration we allowed the BM deposition under normal conditions for the first 3 days and then switched to media containing 8 μg/ml, 30 μg/ml, and 80 μg/ml of the NC1 trimer for the following 3 days (Fig. 4, *D*–*F*, respectively). The well-organized matrix deposited within the first 3 days remained undisturbed, while the newly deposited matrix in the presence of the NC1 trimer revealed concentration-dependent disorganization. The dashed lines in panels D, E, and F (Fig. 4) indicate the visual border between normal and disrupted matrix. The severity of defects increases with the increase of the NC1 trimer concentration in the media. It can be concluded that only the newly forming basement membrane is affected by the recombinant protein.

**Figure 5:**
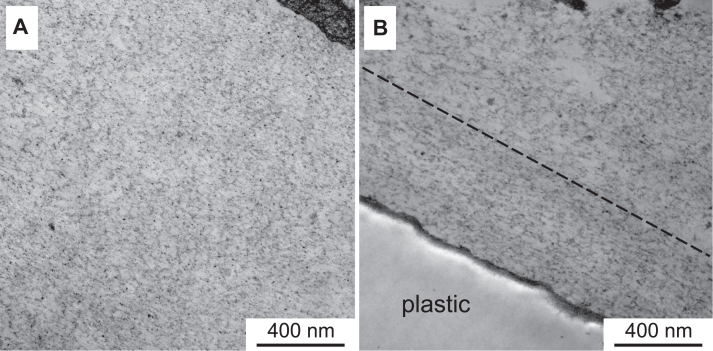
**Trimer but not hexamer disturbs the basement membrane organization.** The PFHR-9 cells were first grown under normal conditions for 3 days, and then either recombinant NC1 hexamer (*A*) or trimer (*B*) was added to the medium for three more days at 80 μg/ml concentration. In (*B*), the *dashed line* indicates the visual border between normal and affected BM structure. The hexamer has no obvious effect on the BM structure (*A*), while trimer (*B*) produces defects identical to those observed in Figure 7.

Since the NC1 domain is also known to be involved in lateral interactions with the protomers (51, 54, 55), we also tested whether the self-assembled NC1 hexamer would have any impact on the BM-like matrix formation. Unexpectedly, the assembled NC1 hexamer does not affect the structure (Fig. 5*A*) while the NC1 trimer causes defects (Figs. 5*B*, 4, *C* and *F*). The dashed line in Figure 5*B* highlights the visible border between normal and disturbed matrix, while the border is indistinguishable in Figure 5*A*. It is thus suggestive that the NC1 trimer to collagen IV protomer oligomerization is an exclusive mechanism disrupting the assembly of the BM. Overall, the NC1 trimer, but not the hexamer, is a potent inhibitor of the BM formation.

### The NC1 trimer can disrupt the collagen IV scaffold

We observed defects in the basement membrane organization upon treating the cell culture with the exogenous NC1 trimer. The ability of the recombinant NC1 trimer to form a complex with the endogenous protomer (see above) suggests interference with the overall collagen IV scaffold assembly by inhibiting endogenous protomer-protomer interactions. To test this hypothesis and find out whether the incorporation of the NC1 trimer into the collagen IV scaffold takes place, the same approach was used. Namely, PFHR-9 cells were grown for 5 days in the presence of 80 μg/ml of either the assembled NC1 hexamer or the NC1 trimer in the growth medium. The medium was replaced daily with a fresh preparation of the NC1 hexamer or trimer to ensure a sufficient supply of the NC1 proteins. In the case of the NC1 trimer, a daily change of the medium was also necessary as it self-assembles into hexamers under high chloride concentration (3, 24, 25, 26) and thus loses the ability to compete with the assembly of endogenous protomers.

**Figure 6:**
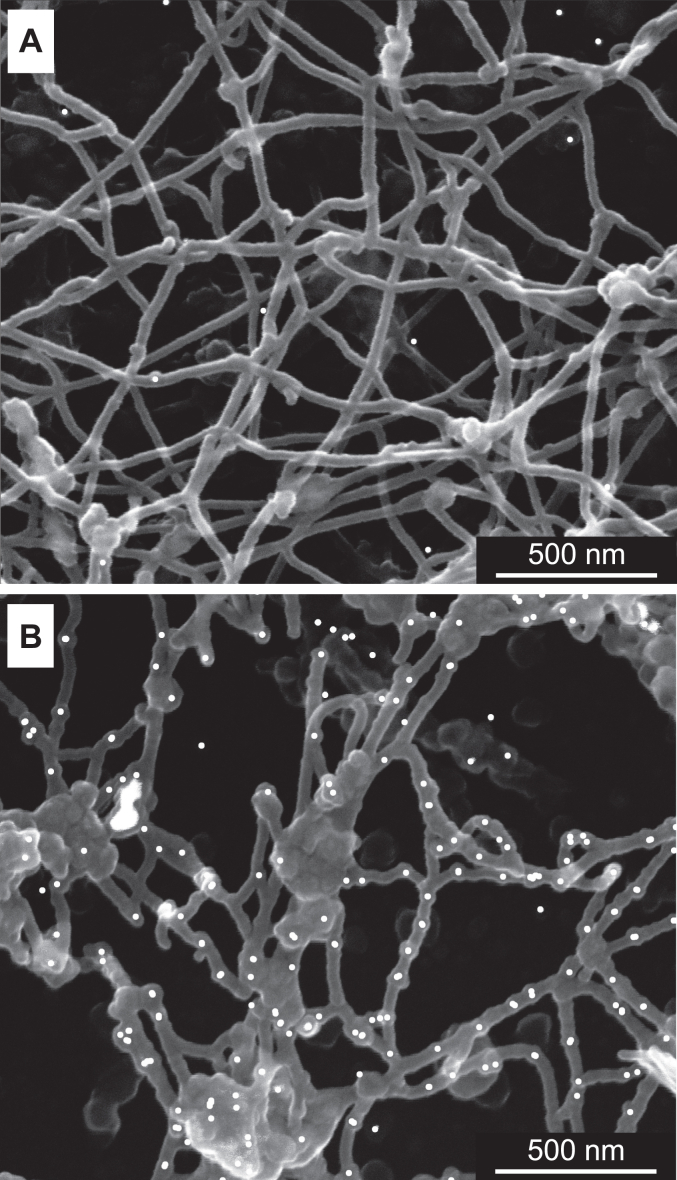
**Incorporation of the recombinant NC1 trimer into the collagen IV scaffold.** Scanning EM of collagen IV scaffolds immuno-labeled against the FLAG-tag found in the recombinant NC1 domain. *A*, cell culture was supplemented with the hexamer assembled from the recombinant NC1 trimer. *B*, cell culture was supplemented with the recombinant NC1 trimer.

**Figure 7:**
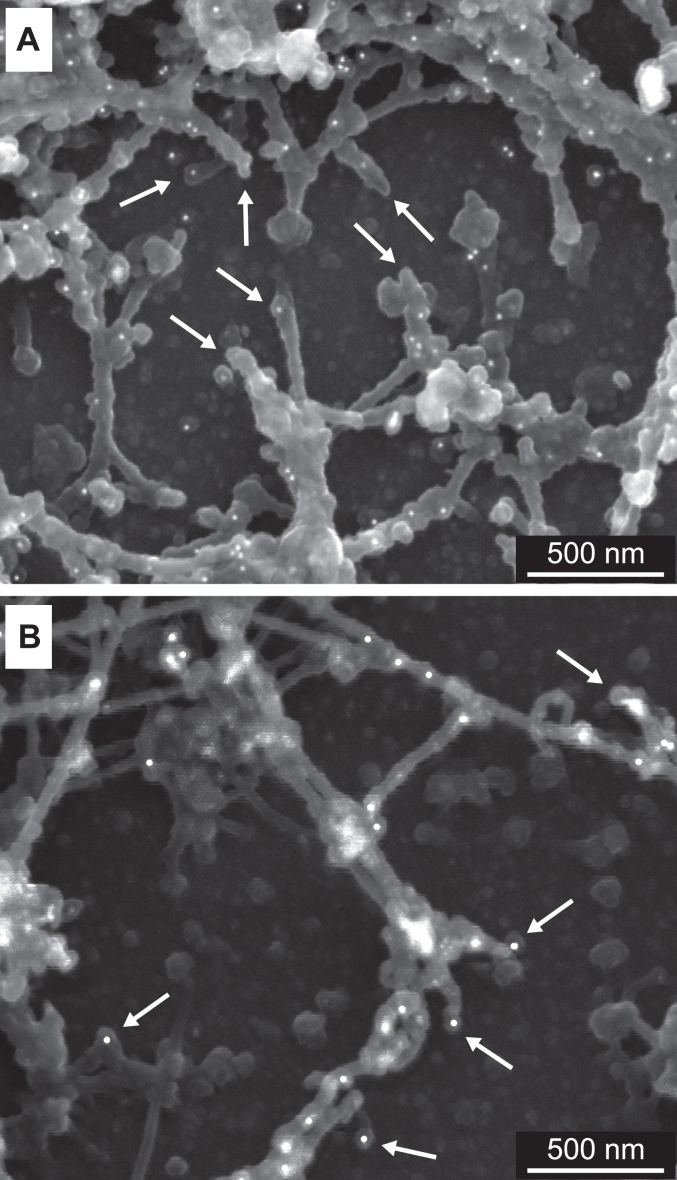
**Disruption of the collagen IV scaffold with the recombinant NC1 trimer.** Immuno-gold scanning EM images of collagen IV scaffolds grown for 5 days in the presence of the NC1 trimer at 80 μg/ml concentration. *A*, sample was immuno-gold labeled with the anti-FLAG antibody, therefore identifying the recombinant single-chain NC1 trimer only. *B*, the sample was labeled using the antibody against the NC1 domain, which recognizes both endogenous and recombinant NC1 domains. A varying degree of scaffold disruption is observed. *White arrows* are pointing to the filament breaks that are capped with the NC1 domain. Binding of the recombinant NC1 trimer along the collagen IV filament is evident.

The resulting scanning EM images of the collagen IV scaffold are represented in Figure 6. The scaffold was immuno-gold labeled with anti-FLAG antibody to detect the incorporation of recombinant NC1 domains. The NC1 trimer was way superior for incorporation in comparison with the hexamer. Notable, the collagen IV scaffold assembled in the presence of the NC1 trimer appeared to be less organized and contained larger cavities (Fig. 6*B*). The scaffolds exhibited defects of varying degrees. By heterotypic hexamer formation (exogenous NC1 trimer to endogenous NC1 trimer of protomer), multiple breaks were observed (Fig. 7). Indeed, the recombinant NC1 trimer competes for the oligomerization with the native NC1 trimers of the endogenous protomers. White arrows point to the filament breaks that are capped with the NC1 trimer.

The binding of recombinant protein along the collagen IV filament is also evident in Figures 6*B*, and 7*B*. Those are recombinant trimers associated with the native NC1 trimers of protomers incorporated into the filaments according to the collagen IV bundle model developed by Yurchenco and Furthmayr (32, 56) from the *in vitro* self-assembly of collagen IV and further confirmed by studies of BM structure in tissues (48, 52, 53).

We did not observe efficient incorporation of the NC1 hexamers into the collagen IV scaffold (Fig. 6*A*), which demonstrated inefficient lateral interactions of the self-assembled NC1 hexamers with the collagenous regions of protomers. Collectively, our results demonstrate that collagen IV scaffold assembly is compromised in the presence of the NC1 trimer. Most notable is the formation of breaks in the continuous filamentous structures where recombinant protein caps the endogenous protomer assemblies. Overall, the NC1 trimer is a potent inhibitor of the collagen IV scaffold assembly, and as we observed above, it translates into disruption of the BM. Thus, the disruption of the collagen IV scaffold cannot be compensated for by other BM components.

### The supplemented NC1 trimer is covalently crosslinked with the endogenous NC1 domain of collagen IV

To verify the heterotypic oligomerization of the NC1 trimer with the protomers within the developing basement membrane, we analyzed the cross-linking pattern of NC1 domains. Model basement membrane was grown in the presence of the NC1 trimer or hexamer at 80 μg/ml concentration in the medium for 5 days. The medium was replaced daily with fresh preparation of the NC1 trimer as described above.

**Figure 8:**
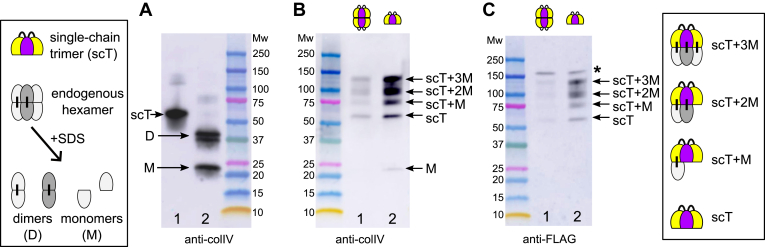
**The NC1 trimer is cross-linked with the endogenous NC1 domain of collagen IV.***A*, schematic representation (*left panel*) and Western blot analysis of the recombinant single-chain NC1 trimer (scT, lane 1) and endogenous NC1 hexamer isolated from the PFHR-9 matrix (lane 2). The hexamer self-assembled *in vitro* from the single-chain NC1 trimer dissociates into trimers under the denaturing conditions of the SDS-PAGE and thus undistinguished from the trimer sample (not shown). The hexamer isolated from the matrix deposited by cells dissociates into monomers (M) and dimers (D) on SDS-PAGE. The primary antibody used was against the NC1 domain. *B*, basement membrane grown in the presence of either assembled hexamer (lane 1) or trimer (lane 2) of the recombinant single-chain NC1 trimer was collagenized to release the NC1 domain, then soluble material was pulled down using anti-FLAG resin and analyzed on Western blot with the same anti-NC1 antibody. *C*, the same as in (*B*) but probed with the anti-FLAG antibody to identify recombinant NC1. ∗ indicates anti-FLAG reactive impurity. This analysis demonstrates that the recombinant NC1 trimer added into the growth media of PFHR-9 cells incorporates into the matrix *via* hexamerization with the endogenous NC1 domain of collagen IV protomer. The crosslinks are formed between the recombinant and endogenous proteins as indicated. The right panel shows all possible crosslinking of the endogenous NC1 domains and the single-chain trimer.

The deposited matrix was subjected to collagenase digest to liberate non-collagenous NC1 domains. Collagenase-solubilized material was immuno-precipitated with the anti-FLAG resin to selectively pull down the recombinant NC1 trimer. Figure 8 shows Western blot analysis of controls (A) and elution fractions after pull-down experiments probed with either anti-NC1 (B) or anti-FLAG (C) antibodies, respectively.

As shown in Figure 8*B*, the NC1 trimer efficiently trapped the endogenous NC1 monomers while the NC1 hexamer tapped only trace amounts (Fig. 8*C*). Additionally, the ladder of bands ranging from NC1 trimer to hexamer with a step of molecular weight corresponding to NC1 monomer (∼23 kDa) was detected, reflecting covalent crosslinking of the recombinant single-chain NC1 trimer with native NC1 monomers (lane 2, Fig. 8*B*). The top bands, but not NC1 monomer and dimer, were also recognized by the anti-FLAG antibody, additionally confirming the recombinant nature of the crosslinked trimer.

Therefore, the model basement membrane grown in the presence of the recombinant single-chain NC1 trimer contains heterotypic NC1 hexamers, which are a hybrid of recombinant and endogenous NC1 domains. The sulfilimine crosslinks are readily formed between the native and recombinant molecules, which emphasizes the functional compatibility of the recombinant protein. In contrast, when added in the hexameric form, only trace amounts of crosslinks were detected with the recombinant NC1 protein in the matrix (lane 1 in Fig. 8, *B* and *C*), possibly due to partial dissociation of the hexamer to trimer.

### The supplemented NC1 trimer does not affect the assembly of 7S tetramers in the collagen IV scaffold

**Figure 9:**
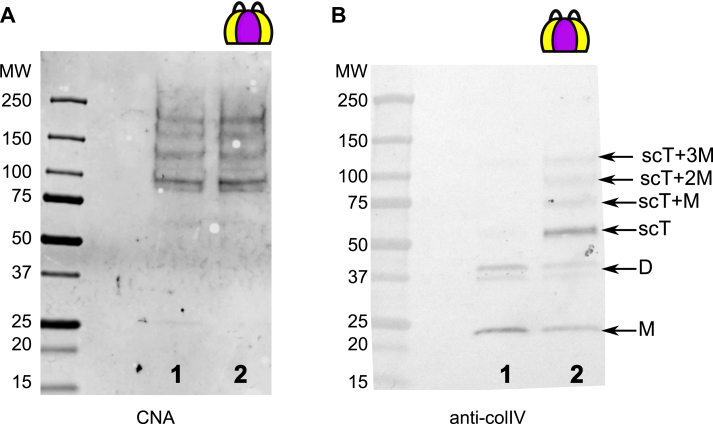
**The NC1 trimer does not affect the formation of the 7S domain in the collagen IV scaffold.** Basement membrane grown under regular conditions (lane 1) or in the presence of 100 μg/ml of the recombinant single-chain NC1 trimer (lane 2) was collagenized to release the 7S and NC1 domains. Samples were run under reducing conditions. The blot was probed with CNA35tri (65) to detect triple helical peptides within the 7S domain (*A*) and anti-colIV antibody to visualize the NC1 domains (*B*).

Our objective was to investigate whether inhibition of collagen IV scaffold assembly through the NC1 domain also influences the assembly of the 7S tetramer. As shown in Figure 9*A*, the presence of the supplemented NC1 trimer does not affect the formation of the 7S tetramer. This indicates that the oligomerization of 7S occurs independently of NC1 and lateral associations. Interestingly, we observed crosslinking of the supplemented trimer with the endogenous NC1, although there was no additional enrichment using the FLAG column. The intensity of the monomers (M) and dimers (D) in the naive matrix is twice as high as in the matrix supplemented with the trimer (Fig. 9*B*). This implies that approximately half of the endogenous NC1 associates with the recombinant material at the provided concentration of the single-chain NC1 trimer.

In summary, the NC1 trimer bears sufficient information for incorporation into the developing BM through native interaction with the NC1 trimer counterpart of the endogenous protomer and crosslinking with it.

## Discussion

There is an unmet need for developing therapies against extracellular matrix-associated congenital and acquired diseases. In the case of collagen IV-associated diseases, such as Alport and Gould syndromes (10, 11, 12, 13, 14, 15, 16, 17, 18, 19, 20, 21, 22), the development of therapies aimed at reconstitution of normal collagen IV function is rational and attractive. To move on, several questions must be addressed, among which targeting and incorporation of a collagen IV substitute are the key ones.

Several studies support the idea of supplementing affected tissue with missing or broken but necessary BM components. Glomerular BM composition and function can be altered *in vivo via* vascular delivery of Laminin-521, the key component of the BM and a culprit of Pierson syndrome (57). In the case of dystrophic epidermolysis bullosa, collagen VII replacement therapy is a promising approach (58, 59). Moreover, rather than full-length molecules, fragments and/or their hybrids may be suitable and efficient for protein replacement therapy in certain cases. Recent examples include the use of mini-agrin and laminin-nidogen hybrid constructs to significantly delay muscular dystrophy in a mouse model (60, 61, 62). The improvement in Alport syndrome through the supplementation of the collagen IV α112 NC1 trimer is unlikely. However, delivering the α345 NC1 trimer may be beneficial in cases where there is a complete absence of the α345 scaffold. This could be especially advantageous if, in future experiments, the NC1 trimer is enhanced with functional collagenous fragments. Conversely, disrupting excessive or misplaced basement membrane formation in pathological conditions, such as diabetic nephropathy and cancer metastasis, may have therapeutic effects. We believe that this approach, a sort of molecular orthotics, aimed at directly fixing the mechanical/architectural/signaling defect of the extracellular matrix (ECM) is a promising direction for developing therapies against multiple congenital and acquired diseases where supramolecular structure and/or function of ECM components is missing, altered, or broken.

We recently discovered that the globular portion of collagen IV in its trimeric form (the NC1 trimer) is targeted to distant tissue locations in *Drosophila* (38). Despite NC1 being only one-seventh part of the whole collagen IV molecule, the NC1 trimer has sufficient information for homing it to the developing BM. This finding provided an initial clue about using NC1-containing fragments of collagen IV for developing protein replacement therapy in humans (39). Here we explored the potential of a globular NC1 domain of collagen IV to be incorporated into developing BM and assess its effects on the collagen IV scaffold and overall structure of BM.

We verified that the recombinant NC1 trimer is functional in forming a complex with the secreted protomers of collagen IV (Fig. 1). We observed an association with the NC1 domain of the endogenous protomer *via* the formation of the NC1 hexamer. We found that it is the NC1 trimer but not the NC1 hexamer that alters the BM structure when supplemented to the medium (Fig. 4). Moreover, it only alters the newly formed BM, while the previously deposited matrix remains undisturbed. Its effect is like the depletion of chloride (a driver for the NC1 hexamer assembly) in the medium as previously reported (25).

At a deeper level of analysis, we found efficient incorporation of the NC1 trimer but not hexamer into the collagen IV scaffold cords (Fig. 6). The NC1 trimer incorporation causes appearance of cord breaks and larger cavities in the scaffold (Fig. 7). Given previous analysis of collagen IV network assembly (52, 53), unraveling of the supercoiled triple helices is possible upon incorporation of the recombinant NC1 trimer. Biochemical analysis revealed that the supplemented NC1 trimer is indeed forming a hexamer complex with the endogenous NC1 domain, which is then crosslinked by sulfilimine bonds (Fig. 8). Notably, this aberrant interaction does not influence the assembly of the endogenous 7S tetramer. Collectively, the NC1 trimer is an active site of collagen IV that is sufficient for efficient incorporation into the growing scaffold of collagen IV. For future developments of active protein replacement formulations, it is imperative to maintain the NC1 domain in its activated, trimeric form for targeting and stable incorporation into the developing BM.

Despite reported interactions of the NC1 hexamer along the collagenous domain of the collagen IV molecule (51, 55) that might contribute to the overall lateral interactions (32, 48, 52, 53, 56) in the scaffold assembly, we found that the NC1 hexamer is an inefficient form for targeting and incorporation.

The NC1 trimer alone is rather detrimental for restoring normal structure and function of altered BM but provides a first clue for targeting and incorporation of designed molecules that should bear additional modules to ensure continuity of the assembling BM, to build required geometry, to form correct suprastructure with other BM components, and to restore functionality.

On the other hand, multiple pathological conditions lead to an excessive formation of the basement membrane. Thus, the ability to interfere with the BM assembly process can be beneficial for inhibiting tumor growth or detrimental angiogenesis and thus has therapeutic potential. Since the collagen IV scaffold is the foundation of the basement membrane, inhibition of its assembly is a promising approach. We observed inhibition of the neoformation of BM in the presence of the NC1 trimer. Thus, the NC1 trimer itself is a potential blocker for proper BM development. Alternatively, the NC1-NC1 trimer assembly into the hexamer is a potential target for screening small-molecule inhibitors.

Finally, heterotypic interaction between the NC1 trimer and the protomer NC1 trimer is a crucial step for targeting and incorporating collagen IV substitutes in several congenital and acquired diseases. Moreover, targeting the NC1-to-NC1 hexamer assembly in the growing tissue might be an efficient way to disrupt BM formation under certain pathologies.

This publication continues a series of papers in the *Journal of Biological Chemistry* that focus on the biology of collagen IV. We conducted an in-depth analysis of animal collagen IV genes across a wide range of species to trace the emergence of six human genes (2). Our research revealed a significant increase in the number of cysteines in a kidney-specific collagen IV isoform, which is likely essential for enduring high hydrostatic pressure in the kidney filter (2). We found that the development of this specialized form has resulted in the compaction of the kidney filter (36). We also established a universal method for the recombinant production of collagen fragments from any type of collagen, using the collagen IV fragment known as CB3 as an example (40). This method opens up exciting possibilities for studying specific mutations, developing supplemental therapies, and solving mysteries related to collagen biology (63). Furthermore, we embarked on an evolutionary investigation to uncover the primordial role of chloride pressure in the assembly and stability of the collagen IV scaffold (24). Our findings underscored the importance of chloride concentration and refined our model for the steps involved in the assembly of the collagen IV scaffold. Using *Drosophila* as a model, we discovered that the globular NC1 domain of collagen IV contains sufficient information to be targeted to a growing basement membrane (38). In this study, we found that the mechanism for NC1 incorporation occurs through the formation of native NC1 hexamers with endogenous NC1 trimers of collagen IV protomers. Additionally, the incorporated NC1 trimer is cross-linked *via* sulfilimine bonds to endogenous NC1 domains. These discoveries contribute to a deeper understanding of collagen IV biology and pave the way for the potential development of novel therapies.

## Experimental procedures

### Cell culture

The PFHR-9 cells (ATCC CRL-2423) were grown to confluency in high glucose (4.5 g/L) DMEM media (Gibco) containing 10% fetal bovine serum (FBS). Production of a basement membrane-like matrix was initiated by the addition of 50 μg/ml of ascorbic acid to ensure hydroxylation in collagen IV and switching to DMEM media with normal glucose (1 g/L).

### Co-assembly of the NC1 trimer with the full-length collagen IV protomers in solution

As was shown in our previous studies, chloride concentration is critical for the collagen IV scaffold formation as it triggers and stabilizes hexamerization of type IV collagen NC1 domains (3, 24, 25, 26). The PFHR-9 cells were grown to confluency, and then the medium was switched to the chloride-free DMEM to ensure the enrichment of trimeric collagen IV protomers in the medium rather than deposition into the matrix. Cells were maintained in the chloride-free media with a daily addition of 50 μg/ml ascorbic acid for 48 h. After 48 h media was collected and 100 μg/ml of the recombinant NC1 trimer was added. This mixture was subsequently dialyzed overnight into the buffer with 150 mM NaCl and then subjected to purification on the anti-FLAG agarose according to the manufacturer’s protocol.

### Rotary shadowing electron microscopy

Samples in phosphate buffer saline (PBS, pH 7.5) were mixed to a final concentration of 70% glycerol, sprayed onto freshly cleaved mica, and rotary shadowed at 6 degrees with a mixture of platinum and carbon in a Balzers BAE 250 evaporator. Replicas were examined in an FEI Tecnai T20 at 120 kV and photographed using an AMT 2X2 camera.

### Generation of a stable PFHR-9 cell line expressing the NC1 trimer

The plasmid pRc-X_ α1α2α1–scNC1 (26) encoding a single-chain NC1 trimer of α1α2α1 composition has been modified to include eGFP sequence to the N-terminus of the NC1 domain by Gibson assembly strategy. The exact scheme and oligos sequences are available upon request. The resulting insert sequence of the pRcX_eGFP-α1α2α1-scNC1 plasmid is also available upon request. The resulting plasmid was sequence-verified and transfected into PFHR-9 cells. Stable clones were isolated using antibiotic selection with G-418 and detection of GFP fluorescence. Individual clones were further selected by analyzing the highest fluorescence signal in conditioned media. Protein expression in conditioned media was also verified by Western blotting. Several clones were selected based on the highest level of expression of eGFP-sc121NC1.

### Protein supplementation to cell culture

In experiments with protein supplementation, the growth media (DMEM at a normal glucose level of 1 g/L) was supplemented with varying concentrations of purified recombinant proteins. The media was replaced every 24 h to provide a fresh source of recombinant proteins.

### Transmission electron microscopy

PFHR-9 cells were seeded and grown to confluency with subsequent matrix deposition on Transwell Permeable Supports (Corning). Varying concentrations of recombinant sc121-NC1 in buffers favoring or preventing hexamerization were added at day 4 and replaced with fresh media through day 6. Supports were fixed in 2.5% glutaraldehyde buffered in 0.1 M sodium cacodylate buffer, pH ∼7.5, post-fixed in 1% osmium tetroxide, followed by dehydration through a graded series of ethanol to 100%. Samples were further dehydrated in propylene oxide and infiltrated and embedded in Spurr’s epoxy. 70-nm ultrathin sections were collected on 300 mesh copper grids and stained in 2% uranyl acetate followed by Reynold’s lead citrate. Stained sections were examined using a T-12 electron microscope (Philips/FEI) operated at 100 kV and photographed using a 2K camera (AMT).

### Scanning electron microscopy

Cells were grown on 13 mm or 22 mm round plastic tissue culture coverslips (Sarstedt, Inc). After matrix deposition was completed, cells were removed by incubation with DOC lysis buffer (10 mM Tris-Cl pH 7.5, 1% sodium deoxycholate, 1 mM EDTA, 0.4 mM PMSF, 10 μg/ml aprotinin, 10 μg/ml leupeptin, and 1 μg/ml pepstatin) for 5 min at room temperature. The lysis buffer was discarded, and the exposed matrix was rinsed once with PBS. To remove most of the BM components the matrix was washed three times with 25 mM EDTA in PBS. The exposed collagen IV scaffold was briefly washed with EM grade water and fixed in 2.5% glutaraldehyde buffered in 0.1 M sodium cacodylate buffer, pH ∼7.5 either overnight in the cold room for standard scanning EM or 15 min at room temperature for immuno-gold labeled scanning EM. Different fixation protocols, including no fixation, were tried for immuno-gold labeling experiments. Fixation with 2.5% glutaraldehyde for 15 min prior to the primary antibody produced almost no background labeling while still yielding targeted labeling. Samples went through the standard protocol for scanning EM preparation including post-fixation in 1% osmium tetroxide (reduced to 20 min for immuno-gold samples), serial dehydration, and critical point drying (Samdri-PVT-3D, Tousimis). Finally, samples were either sputter coated with platinum/gold (Cressington 108 sputter coater) for 30 s or carbon coated for 1 s (Carbon coater, Electron Microscopy Sciences) for immuno-gold samples. Images were taken with Quanta 250 Environmental Scanning Electron Microscope in secondary and backscattering modes. For immuno-gold labeled samples the images produced by secondary and backscattered electrons were overlaid and gold particles were artificially enhanced in the GIMP program.

#### Distances calculations from immuno-gold images

The number of gold particles per 400 nm length was calculated as the whole number of gold particles in the image view divided by the total fibril length and multiplied by 400 nm. The total fibril length was calculated by Angiogenesis Analyzer package for the ImageJ software. The Nearest Neighbor Distance (NND) was calculated using plugin for ImageJ (64).

#### Antibodies used for Western blot analysis and immuno-gold labeling

For immuno-gold scanning EM, the primary mouse monoclonal antibodies were against human NC1 domain of type IV collagen (in-house mouse antibody; mab 1547) or anti-FLAG M2 antibody (Sigma-Aldrich). A secondary 15 nm gold conjugated goat anti-mouse antibody (Electron Microscopy Sciences) was used for scanning EM. Western blotting was done using rat anti-collagen IV NC1 antibody (1:250 dilution, JK2; from Y. Sado, Shigei Medical Research Institute, Okayama, Japan) or anti-FLAG M2 antibody (Sigma-Aldrich).

## Data availability

All data are contained within the manuscript. Raw SEM images from this study are available by emailing the corresponding author.

## Conflict of interest

The authors declare that they have no conflicts of interest with the contents of this article.
